# Ureteral orifice edema and stenting challenges in diabetic patients: Lessons from a case report

**DOI:** 10.1097/MD.0000000000042069

**Published:** 2025-04-04

**Authors:** Shuang Guo, Yunxi Hu, Zhongwei Liu, Denghui Huang, Wenjiang Yang

**Affiliations:** a Urological Surgical Department, Wenshang County People’s Hospital, Jining, Shandong, China; b Wenshang County People’s Hospital, Jining, Shandong, China; c Surgical Teaching and Research Office, Wenshang County People’s Hospital, Jining, Shandong, China.

**Keywords:** diabetes, inflammation, ureteral calculus, ureteral stent

## Abstract

**Rationale:**

Ureteral stones, particularly in elderly patients, pose significant clinical challenges due to their association with severe pain, infection, and urinary obstruction. While the introduction of minimally invasive techniques, such as ureteroscopy and ureteral stenting, has revolutionized treatment, gaps remain in understanding how comorbid conditions like diabetes affect patient outcomes.

**Patient concerns:**

A 68-year-old male patient was admitted to our hospital for 3 days due to left lumbar and abdominal pain accompanied by fever.

**Diagnoses:**

Abdominal computed tomography revealed sediment calculi in the left lower ureter and significant exudative changes in the left kidney. Through laboratory examination, procalcitonin and other inflammatory indicators were significantly elevated. The diagnosis was ureteral calculi with infection.

**Interventions:**

Transurethral ureteral stenting.

**Outcomes:**

The patient was discharged 3 days after surgery, and the inflammation index returned to normal. The ureteral stent was removed at a 2-week follow-up.

**Lessons:**

Key findings include the identification of significant challenges in locating the ureteral orifice, the successful use of stenting to manage both infection and obstruction, and the importance of minimally invasive interventions in diabetic patients. The case also emphasizes the role of diabetes in complicating ureteral stone management due to its impact on immune response and healing. This report contributes to the existing literature by providing insights into the complex anatomical and pathological factors that can complicate ureteral stent placement in diabetic and elderly patients.

## 1. Introduction

Ureteral stones represent a common cause of hospital admissions, particularly in the elderly population, and present notable clinical challenges due to their potential to induce severe pain, infection, and urinary tract obstruction.^[1]^ The management of ureteral stones has undergone substantial evolution over recent decades, shifting from traditional open surgical approaches to minimally invasive techniques such as ureteroscopy, extracorporeal shock wave lithotripsy, and percutaneous nephrolithotomy.^[2]^ These advancements have markedly enhanced patient outcomes and shortened recovery periods, positioning endourological techniques as the foundational approach in contemporary ureteral stone treatment.^[3]^

In the initial phase of ureteral stone research, open surgery was the predominant method of treatment.^[4]^ The introduction of ureteroscopy in the 1980s marked a significant advancement, enabling direct visualization and extraction of stones through minimally invasive techniques.^[5]^ This method, combined with the development of ureteral stenting, has considerably reduced the morbidity associated with surgical stone management while maintaining high stone clearance success rates.^[6]^ Ureteral stent placement has become a routine procedure for managing ureteral obstruction, alleviating symptoms, and preventing infection-related complications such as pyelonephritis and urosepsis.^[7]^

Key studies, particularly those examining the efficacy of various endoscopic interventions, have provided critical insights into the optimal management of ureteral stones, especially in complex cases involving obstruction, infection, or anatomical abnormalities. These investigations have confirmed the use of stents as a vital measure in minimizing complications and promoting postoperative recovery.^[8]^ Further research has explored the role of stenting in patients with recurrent stone formation or comorbid conditions such as diabetes, highlighting the necessity of individualized treatment strategies.^[9]^

Despite these advancements, significant gaps persist in the literature, particularly concerning the long-term outcomes of stent placement in elderly patients with comorbidities such as diabetes. Existing research has predominantly concentrated on immediate postoperative recovery, leaving unresolved questions about the long-term effects of stent retention, its impact on kidney function, and the risk of recurrent stone formation in high-risk populations.^[10]^

The case report discussed in this study, involving a 68-year-old diabetic male with left ureteral stones and associated hydronephrosis, underscores the complexities involved in managing such cases. Challenges in locating the ureteral orifice due to edema and inflammation, alongside the successful stent placement following cystoscopy, emphasize the necessity for further research into optimal stent management practices for similar patients. This study specifically aims to investigate postoperative outcomes, the recurrence of stone formation, and the influence of comorbidities such as diabetes on ureteral stone treatment. By addressing these gaps, the research will enhance the understanding of long-term stent management in elderly and diabetic populations, ultimately informing improvements in clinical practice.

## 2. Case report

A 68-year-old male patient was admitted to the hospital due to left-sided abdominal and lumbar pain accompanied by fever for 3 days. The pain began without any apparent cause, presenting as continuous left abdominal and lumbar pain, accompanied by fever and chills. The patient self-reported a maximum temperature of 38.5 °C, along with nausea and a single episode of non-projectile vomiting consisting of gastric contents. He denied coughing, sputum production, frequent urination, urgency, dysuria, or gross hematuria. His medical history was significant for a 2-year history of diabetes mellitus. Physical examination revealed deep tenderness in the left lower abdomen without rebound tenderness, and significant left costovertebral angle tenderness.

**Figure 1.**
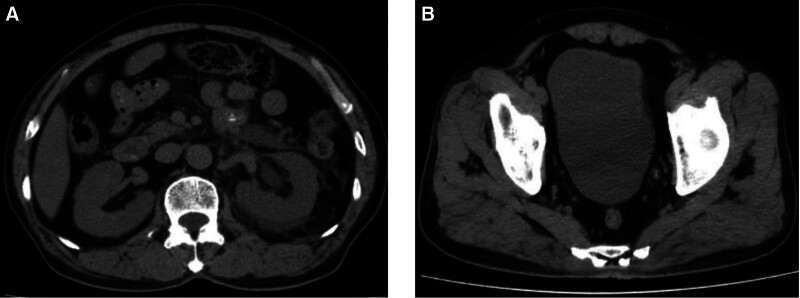
Abdominal computed tomography. (A) Exudative changes in the left kidney. (B) Sediment calculi changes in the left lower ureter.

Laboratory tests revealed an elevated white blood cell count of 12 × 10^12^/L, positive nitrite detection in urine, 3 + leukocytes, 3 + urine glucose, a procalcitonin level of 1.3 ng/mL, an erythrocyte sedimentation rate of 40 mm/h, and a C-reactive protein level of 50 mg/L. The glycated hemoglobin was 10.4%. Blood glucose levels remained above 20 mmol/L. Abdominal computed tomography scan revealed sand-like stones in the lower left ureter and moderate hydronephrosis of the left kidney (Fig. 1A and B).

**Figure 2.**
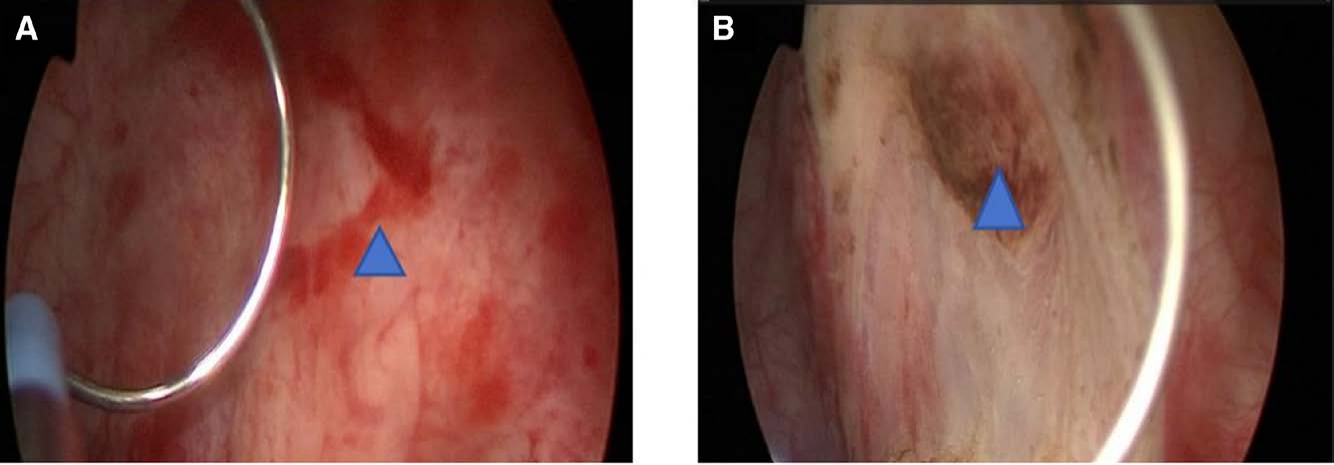
Field of view under ureteroscope. (A) Position of the left ureteral opening (indicated by triangular star) before electrotomy. (B) Position of the left ureteral opening after electrotomy (indicated by triangular star).

Upon admission, the patient was initiated on anti-inflammatory therapy, but his body temperature continued to spike intermittently. Consequently, a transurethral ureteral stent placement was performed. Intraoperatively, the right ureteral orifice was located easily, with normal urine jetting from the right side. However, the left ureteral orifice was difficult to locate, and the area surrounding it appeared edematous. After filling and draining the bladder, attempts to locate the left ureteral orifice were still challenging. Gentle insertion of a guide wire or ureteral catheter resulted in the release of some sand-like material, suggesting the location of the ureteral orifice. Cystoscopy was performed, and after resection of the edematous area, the pus-filled ureteral orifice was exposed. A ureteral stent was successfully placed through this orifice (Fig. 2A and B).

**Figure 3.**
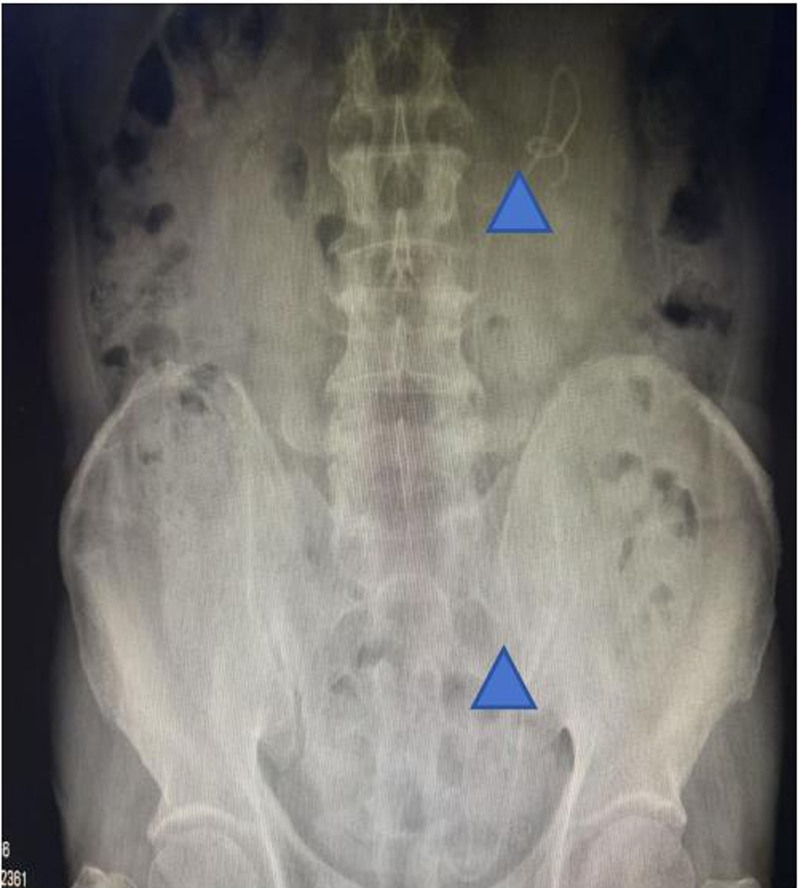
Abdominal radiograph, ureteral stent position (indicated by triangular star).

**Figure 4.**
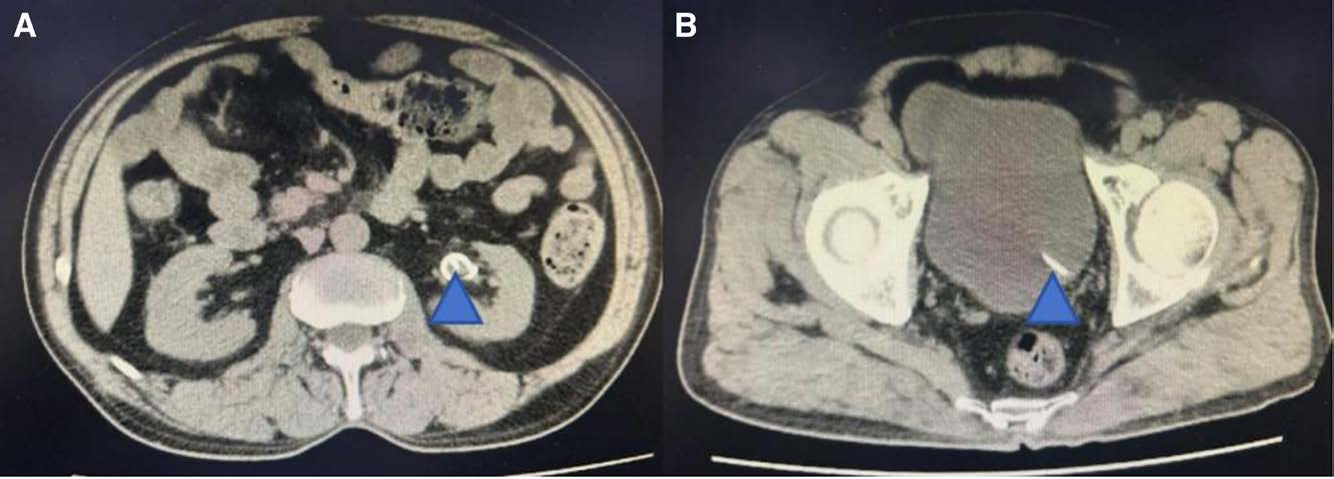
Abdominal computed tomography. (A) The ureteral stent is located in the renal pelvis (indicated by triangular star). (B) The ureteral stent is located in the bladder (indicated by triangular star).

Postoperatively, the patient’s vital signs remained stable, and an abdominal anteroposterior radiograph indicated normal positioning of the ureteral stent (Fig. 3). Anti-inflammatory therapy was continued, and blood glucose levels were monitored. The patient’s blood glucose levels before and after each meal, as well as at bedtime, had decreased significantly to around 15 mmol/L. Following consultation with an endocrinologist, insulin therapy was administered to stabilize the patient’s blood glucose levels within the normal range. The patient was discharged 3 days after the surgery, and inflammatory markers returned to normal upon reexamination. During the follow-up period, 2 weeks postoperatively, a full abdominal computed tomography scan was performed, showing normal positioning of the ureteral stent (Fig. 4A and B), which was then removed.

## 3. Discussion

This case report features a 68-year-old diabetic male patient presenting with ureteral stones, significant inflammation, and hydronephrosis, successfully managed through the placement of a ureteral stent. The importance of this case lies in its demonstration of the complexities involved in treating ureteral stones in elderly patients with comorbidities such as diabetes, a population that frequently poses unique challenges in both diagnosis and treatment.

The successful management of this patient’s condition underscores the critical role of timely and minimally invasive interventions in cases where ureteral stones lead to both obstruction and infection. This case further contributes to the expanding body of evidence supporting the use of ureteral stents, not only for immediate symptom relief but also for the prevention of complications such as urosepsis. Additionally, it enhances understanding of how diabetes—a condition that impairs immune response and healing—can complicate the management of urinary tract conditions, including ureteral stones.

In the broader context of contemporary research, this case highlights the significance of tailored treatment strategies for high-risk populations. Although advanced methods such as ureteroscopy and stent placement are well-established in stone management, the complexities associated with comorbidities like diabetes necessitate further investigation to enhance treatment efficacy. This case emphasizes that, in elderly and diabetic patients, a proactive approach incorporating imaging, laboratory diagnostics, and minimally invasive surgical interventions can achieve favorable outcomes, even when complications such as inflammation and infection are present.

The chronic hyperglycemia state in diabetic patients can exacerbate the risk of infection through various pathways. For instance, advanced glycation end products inhibit chemotaxis, phagocytosis, and bactericidal functions.^[11]^ Hyperglycemia impairs urinary tract mucosal defense by promoting pathogen adhesion.^[12]^ Microcirculation disorders, such as diabetic microvascular disease, can reduce antibiotic tissue permeability by 30% to 40%.^[13]^ Systemic inflammatory responses like fever may be masked by hyperglycemia, increasing the risk of rapidly progressing to emphysematous pyelonephritis by several times.^[14]^ The incidence of sepsis is several times higher in diabetic patients compared to non-diabetes mellitus patients, with delayed treatment response and prolonged time to achieve antibiotic treatment targets.^[15]^ On the other hand, diabetes amplifies the presence of inflammatory responses. Due to comorbid diabetes, the hyperglycemic environment significantly exacerbates the degree of local inflammation and edema. Studies have shown that diabetic patients have reduced ureteral mucosal repair capacity due to microvascular disease and immunosuppression, resulting in a 2.3 times higher failure rate of stent placement compared to nondiabetic patients.^[16,17]^

The findings from this case are consistent with the current literature on the effectiveness of ureteral stents in managing obstructive uropathy and mitigating postoperative complications.^[16]^ Previous studies have shown the value of stenting in alleviating ureteral obstruction, particularly in cases complicated by infection or other comorbid conditions.^[18]^ Additionally, research involving diabetic patients with urolithiasis has underscored the heightened risk of infection and the necessity for vigilant postoperative monitoring, as demonstrated in this case.

What distinguishes this case is the challenging nature of identifying the ureteral orifice due to edema and inflammation, requiring a more meticulous and refined approach to stent placement. Although ureteral stenting is a routine procedure, the complexity encountered in this instance emphasizes the variability in clinical presentations and highlights the necessity for further investigation into the anatomical and pathological factors that can complicate such interventions. This case offers important insights into the critical role of real-time surgical decision-making, such as utilizing cystoscopy to locate the ureteral orifice, in managing complex cases effectively.

While this case report provides important insights into the management of ureteral stones in diabetic and elderly patients, it has several limitations. Firstly, as a single case study, its findings lack the generalizability required to draw broad conclusions about the entire population of elderly or diabetic individuals with urolithiasis. Moreover, the absence of long-term follow-up data limits the ability to assess the recurrence of stone formation, the patient’s renal function over time, and potential complications related to stent retention.

Another limitation is the lack of comparative data on alternative treatment modalities, such as extracorporeal shock wave lithotripsy or percutaneous nephrolithotomy, which could be more suitable for certain patient populations. Although stent placement proved successful in this case, it is essential to recognize that other techniques may offer distinct benefits or present different risks, particularly in patients with varying anatomical factors or stone compositions.

The outcomes of this case point to several directions for future research. A primary area of interest is the long-term effects of ureteral stenting in elderly diabetic patients, specifically regarding stone recurrence and renal function. Longitudinal studies that follow patients after stent removal could offer valuable insights into how comorbid conditions affect recovery and the risk of subsequent stone formation.

Additionally, further research is necessary to investigate the specific anatomical challenges encountered in patients with severe ureteral inflammation or edema. This case illustrates the complexities of stent placement under such conditions, highlighting the potential need for advanced imaging techniques or novel surgical tools that could improve success rates in similar situations.

## 4. Conclusion

Lastly, research should prioritize the refinement of postoperative care protocols for diabetic patients, who face an increased risk of infection and delayed recovery. Establishing targeted guidelines for postoperative monitoring and intervention in this population could reduce complication rates and enhance overall outcomes.

## Acknowledgments

We thank the patient and his family for their cooperation.

## Author contributions

**Data curation:** Shuang Guo, Denghui Huang.

**Formal analysis:** Shuang Guo, Zhongwei Liu.

**Funding acquisition:** Shuang Guo.

**Investigation:** Shuang Guo.

**Methodology:** Zhongwei Liu, Denghui Huang.

**Resources:** Zhongwei Liu.

**Software:** Yunxi Hu.

**Supervision:** Yunxi Hu, Denghui Huang.

**Validation:** Yunxi Hu.

**Visualization:** Yunxi Hu.

**Writing—original draft:** Wenjiang Yang.

**Writing—review & editing:** Wenjiang Yang.
